# Montelukast: risk of mental disorders vs. efficacy–a meta-analysis

**DOI:** 10.3389/fphar.2025.1659852

**Published:** 2025-10-24

**Authors:** Marharyta Sobczak, Rafał Pawliczak

**Affiliations:** Department of Immunopathology, Medical Faculty, Medical University of Lodz, Lodz, Poland

**Keywords:** montelukast, asthma, allergic rhinitis, efficacy, suicide

## Abstract

**Background:**

Montelukast, a selective leukotriene receptor antagonist, is widely used in the treatment of bronchial asthma and allergic rhinitis (AR). Although it is a well-tolerated drug, there are reports of possible central nervous system side effects, including, for example, mood changes and suicidal thoughts. Therefore, we conducted a meta-analysis to test the effects of montelukast on the mental health of patients taking montelukast and to test its effectiveness in treating asthma and AR.

**Methods:**

PubMed, Web of Science, and the Cochrane Central Register of Controlled Trials databases were searched to find articles of control-compared randomized clinical trials, which investigated the efficacy of montelukast treatment as well as articles about mental disorders after this treatment. The relative risk with 95% confidence interval (CI) and the standardized mean difference with 95% CI were calculated to compare the effect. A random effects model was used to calculate effect sizes.

**Results:**

Our meta-analysis was based on 4 studies (mental health analysis) and 19 studies (efficacy analysis). We indicated that montelukast treatment was associated with a higher risk of anxiety by 11% (RR = 1.11; 95% CI [1.06; 1.16]; p < 0.0001, I^2^ = 0%) without differences between subgroups. Meta-analysis showed the different efficacy of montelukast against asthma and AR symptoms in comparison to placebo and other drugs.

**Conclusion:**

In terms of treating asthma and allergic rhinitis, montelukast shows comparable efficacy to other drugs, such as inhaled corticosteroids or second-generation antihistamines. Furthermore, montelukast was associated with a modestly increased risk of anxiety, while no consistent evidence was found for an increased risk of depression or suicidal behaviors, considering the limited data.

## 1 Introduction

Asthma and allergic rhinitis (AR) are disorders that affect a large part of the population. These two conditions often occur together in affected individuals, sharing a common pathophysiology. While asthma is a chronic inflammatory disease of the airways, manifested by a variety of symptoms related to inflammation and airway hyperresponsiveness, AR can manifest as rhinorrhea, enlargement of the nasal auricles and their tenderness and conjunctival congestion (Zuberi et al., 2020). Allergic rhinitis has traditionally been classified as seasonal or perennial, depending on the temporal pattern of symptoms. Although there are some people who are allergic to seasonal and perennial allergens at the same time. What’s more, AR is a strong risk factor for asthma, with as many as 81% of patients with asthma reporting experiencing rhinitis symptoms (Siddiqui et al., 2022).

Cysteinyl leukotrienes (CysLTs) are endogenous inflammatory mediators that play a crucial role in allergic airway diseases by stimulating bronchoconstriction, mucus production, mucosal swelling and inflammation, airway infiltration by eosinophils and maturation of dendritic cells, which prepares them for a future allergic response. Montelukast inhibits these actions by blocking CysLT1 receptors located on immunocytes, smooth muscle and endothelium of the airway mucosa (Nayak and Langdon, 2007). Montelukast is a drug introduced in 1998 for use in the United States and is currently used to treat asthma and AR (Zuberi et al., 2020). Interestingly, montelukast has a similar safety and tolerability profile to placebo during both short-term and long-term administration (Amlani et al., 2011). However, in 2009, Food and Drug Administration (FDA) reviewed clinical trial data on reports of aggression, hallucinations, depression, insomnia and suicidal thoughts in association with leukotriene receptor antagonists use (Drug Safety Information for Heathcare Professionals - Updated Information on Leukotriene Inhibitors, 2025). Later, in 2020, the FDA issued a black box warning about montelukast’s negative effects on mental health (Lo et al., 2023). Unfortunately, such adverse events are rare and difficult to detect in randomized controlled trials (RCTs) with short follow-up periods and relatively small sample sizes (Law et al., 2018). According to a cross-sectional study, montelukast also affects sleep disorders (Zhu and Lv, 2025). It is worth noting that prolonged use of montelukast in older children (aged 6–15 years) may lead to an elevated risk of developing Tourette syndrome or tics (Lei et al., 2025). On the other hand, however, montelukast has demonstrated neuroprotective effects via antioxidant, anti-inflammatory, and anti-apoptotic mechanisms. Montelukast has been shown to have neuroprotective effects in several neuronal diseases, such as Alzheimer’s disease, cerebral ischemia, multiple sclerosis and seizures (Tesfaye et al., 2021). Due to conflicting evidence regarding the effects of montelukast on the nervous system, we conducted a meta-analysis to test the effects of montelukast on the mental health of patients taking montelukast and to test its effectiveness in treating asthma and AR.

## 2 Methods

### 2.1 Search strategy

This meta-analysis was conducted according to the Preferred Reporting Items for Systematic Reviews and Meta-Analyses (PRISMA) guidelines (Moher et al., 2015). PubMed, Web of Science and the Cochrane Central Register of Controlled Trials databases were searched to find literature published before February 9, 2025, using defined keywords combined with Boolean operators. The following keywords were used for psychiatric disorder analysis: *“Montelukast”, “Montelukast sodium”, “behavioral disorders”, “mental disorders”, “suicide”, “psychiatric disorders”;* and for efficacy analysis: *“Montelukast”, “Montelukast sodium”, “asthma”, “allergic rhinitis”, “efficacy”, “effectiveness”.* PubMed used automatic mapping, Web of Science used “All Fields”, and Cochrane Library used ‘Advanced Search’. No filters or language restrictions were applied during the search.

Additionally, we used data from public available databases, such as FDA Adverse Events Reporting System (FDA, 2025) (up to March 21, 2025), EudraVigilance - European database of suspected adverse drug reaction reports (up to May 11, 2025) (Adrreports, 2025) and the Medicines and Healthcare products Regulatory Agency (MHRA) (MHRA, 2025) (up to May 14, 2024).

### 2.2 Study selection and data extraction

Inclusion criteria for analysis of mental disorders included all articles, which contain data on mental disorders after treatment with montelukast, such as number of patients with anxiety, number of patients with depression and number of patients with suicidal and self-injurious behaviors.

Inclusion criteria for efficacy analysis included only articles of RCTs that investigated the efficacy of montelukast to treatment asthma and AR, were written in English and containing the following outcomes: changes in peak expiratory flow rate (PEF/PEFR) in the morning and in the evening in patients with asthma; changes in daytime symptoms, such as total nasal symptoms or daytime composite symptoms, nasal congestion or nasal obstruction, nasal itching or nasal pruritus and sneezing in patients with asthma and AR, congestion, itching or pruritus, rhinorrhea and sneezing in patients with AR, changes in daytime asthma symptoms in patients with asthma; changes in nighttime symptoms, such as total nasal symptoms or nighttime composite symptoms and nasal congestion or nasal obstruction in patients with asthma and AR, nasal congestion on awakening, difficulty getting to sleep due to nasal symptoms and nighttime awakenings due to nasal symptom in patients with AR and changes in nighttime asthma symptoms in patients with asthma; and changes in daily nasal symptoms, such as sneezing, rhinorrhea or runny nose, nasal congestion or stuffy nose in patients with AR.

We excluded studies about exercise-induced asthma and cough-variant asthma. We also excluded studies incompatible with the treatment scheme: montelukast vs. placebo, montelukast vs. drug and montelukast + drug vs. drug. M.S. was responsible for the process of selecting studies eligible for meta-analysis. The study selection process was conducted according to the PRISMA flow guidelines (Supplementary Figure S1; Supplementary Figure S2).

Continuous data was converted into mean and standard deviation [mean (SD)].

• If the data was presented as a median with quartiles [median (Q1, Q3)] the value was converted according to method presented by Luo et al. (Luo et al., 2018) and Wan et al. (Wan et al., 2014) using available calculator without checking the skewness.
• If the data was presented as a mean (95% confidence intervals), the value was converted according to Cochrane Handbook for Systematic Reviews of Interventions (Higgins et al., 2023) using the formula: $SD=\sqrt{N}\times \left(upper\;limit-lower\;limit\right)/3.92$

• If the data was presented as a mean (range), the value was converted according to Wan et al. (Wan et al., 2014).

### 2.3 Quality assessment

The quality of trials was assessed using the Cochrane Collaboration’s tool for assessing risk of bias in randomized trials (Higgins et al., 2011). The following criteria were used: random sequence generation, allocation concealment, blinding of participants and personnel, blinding of outcome assessment, incomplete outcome data, selective reporting and other bias (assessed at 3 levels such as low, high or unclear risk).

### 2.4 Statistical analysis

Statistical analysis of the data was performed in R (version 4.2.2). To compare the effect of montelukast treatment in the experimental group compared to the control group, the standardized mean difference with 95% CI was calculated for continuous outcomes, while the relative risk (RR) with 95% confidence interval (CI) for dichotomous outcomes. Random effects model was used to calculate effect sizes and to account for expected heterogeneity across studies in terms of design, populations, and outcome measures. I^2^ statistic was used to evaluate the heterogeneity of studies: I^2^ < 40% may not be important; 30% < I^2^ < 60% means moderate heterogeneity; 50% < I^2^ < 90% means substantial heterogeneity; I^2^ > 75% means considerable heterogeneity (Deeks et al., 2008). Funnel plot, Peters’ regression test (for dichotomous outcomes) and Egger’s regression test (for continuous outcomes) were used to assess publication bias. The results of this meta-analysis were considered statistically significant at p < 0.05.

## 3 Results

### 3.1 Search results

For mental disorders analysis, literature search yielded 112 articles after removal of duplicates (Supplementary Figure S1). In the first screening, we excluded 81 articles, such as meta-analysis, systematic reviews, literature reviews, editorial letters, as well as *in vitro* studies, studies on animals and case reports. After full-text screening, 4 articles were qualified for meta-analysis. These articles are retrospective analysis, which were conducted on Korea Adverse Event Reporting System (Shin et al., 2024) from 2014 to 2018, the TriNetX Analytics Network patient repository (Paljarvi et al., 2022) from 2015 to 2019, Merck clinical trial data (Philip et al., 2009) completed by April 25, 2008 and the FDA Adverse Events Reporting System (Schumock et al., 2012) from 1999 to 2009. Psychiatric outcomes in the included studies were identified using various coding systems, including the Medical Dictionary for Regulatory Activities (MedDRA) (Philip et al., 2009; Schumock et al., 2012), the World Health Organization-Adverse Reaction Terminology (WHO-ART) (Shin et al., 2024), and International Statistical Classification of Diseases, Tenth Revision, Clinical Modification (ICD-10-CM) codes (Paljarvi et al., 2022).

For efficacy analysis, literature search yielded 2291 articles after removal of duplicates (Supplementary Figure S2). In the first screening, we excluded 1960 articles, such as meta-analysis, systematic reviews, literature reviews, editorial letters, as well as *in vitro* studies, studies on animals, case reports and observational studies. After full-text screening, 19 articles were qualified for meta-analysis. All included studies are randomized controlled trials investigated the efficacy of montelukast to treatment asthma or/and allergic rhinitis. These studies were carried out in the United States of America (United States) (Busse et al., 2006; Nathan et al., 2005; Martin et al., 2006; Esteitie et al., 2010; Ratner et al., 2003; Szefler et al., 2007; Calhoun et al., 2001; Meltzer et al., 2002; Pearlman et al., 2002; Busse et al., 2001; Noonan et al., 1998), China (Li et al., 2009), the United States of America and Europe (Philip et al., 2004), the United States of America and Puerto Rico (Fish et al., 2001), Turkey (Razi et al., 2006; Yurdakul et al., 2003), Taiwan (Chen et al., 2006) and Germany (Kanniess et al., 2002), India (Shah et al., 2006). Some studies included only pediatric patients (Szefler et al., 2007; Li et al., 2009; Razi et al., 2006; Chen et al., 2006). Table 1 presents the characteristics of the studies included.

**TABLE 1:** Basic characteristics of included studies.

Studies	Study design	Participants	Mean age (mean, SD)	Sex (girl/female)	Treatment	Duration of the montelukast treatment
Busse et al. (2006)	a randomized, double-blind, parallel-group study	18 years or older patients with chronic asthma and seasonal aeroallergen sensitivity	I: 35.5 (12) C: 36.8 (14.5)	I: 72.4% C: 67.8%	I: montelukast 10 mg once daily C: placebo	3 weeks
Nathan et al. (2005)	a randomized, double-blind, parallel-group study	at least 15 years of age patients with a history of seasonal allergic rhinitis and had a diagnosis of persistent asthma	I: 34.4 (13.3) C: 35.7 (14.0)	I: 66% C: 72%	I: montelukast 10 mg once daily + fluticasone propionate/salmeterol 100/50 µg twice daily (plus vehicle placebo for aqueous nasal spray) C: fluticasone propionate/salmeterol 100/50 µg twice daily (plus placebos for both active treatments)	4 weeks
Li et al. (2009)	a double-blinded, randomized, placebo-controlled trial	6–18 years old children with stable asthma and persistent allergic rhinitis	I: 11.9 (3.2) C: 12.3 (3.2)	I: 45.5% C: 63.6%	I: montelukast (5 mg for <14 years or 10 mg for 14 years) + fexofenadine (60 mg for <12 years and 120 mg for 12 years) C: placebo + fexofenadine (60 mg for <12 years and 120 mg for 12 years)	16 weeks
Philip et al. (2004)	a randomized, parallel-group, double-blind, double-dummy study	15–85 years old patients with clinical history of active asthma and seasonal allergic rhinitis	I: 33.0 (13.2) C: 33.6 (13.7)	I: 63.9% C: 64.7%	I: montelukast 10 mg daily C: placebo	2 weeks
Martin et al. (2006)	a multicenter, double-blind, double-dummy, randomized, parallel-group study	at least 15 years old patients with the seasonal allergic rhinitis	I: 40.3 (13.9) C: 39.1 (14.0)	*NA*	I: montelukast 10 mg daily (plus matched vehicle placebo for aqueous nasal spray) C: fluticasone propionate 200 µg daily (plus matched placebo for montelukast)	2 weeks
Razi et al. (2006)	a randomized, double-blind, parallel-group study	7–14 years old patients with seasonal allergic rhinitis with exacerbations during the spring pollen season	I: 11.50 (0.89) C: 11.42 (0.67)	I: 38% C: 50%	I: montelukast 5 mg once daily C: placebo	2 weeks
Esteitie et al. (2010)	a 4-week parallel, randomized, double-blind, placebo-controlled trial	18–55 years old patients with symptoms of perennial allergic rhinitis	I: 35 (10.47) C: 32 (9.63)	I: 71% C: 57.7%	I: montelukast 10 mg + fluticasone propionate 200 µg daily C: placebo + fluticasone propionate 200 µg daily	2 weeks
Ratner et al. (2003)	a multicenter, double-blind, double-dummy, randomized, parallel-group study	at least 15 years of age, resided in south central Texas where the mountain cedar allergen is prevalent, and had a diagnosis of seasonal allergic rhinitis	I: 38.1 (13.3) C: 38.3 (13.3)	I: 63% C: 61%	I: montelukast 10 mg once daily (plus matched vehicle placebo for fluticasone propionate) C: fluticasone propionate aqueous 200 µg once daily (plus matched placebo for montelukast)	15 days
Chen et al. (2006)	a randomized, double-blind, placebo-controlled, parallel-group study	2–6 years old children with a clinical history of perennial allergic rhinitis	I: 4.49 (1.09) C1: 4.53 (0.91) C2: 4.36 (0.87)	I: 45% C1: 40% C2: 55%	I: montelukast 4 mg daily C1: cetirizine 5 mg daily C2: placebo 5 mg daily	12 weeks
Yurdakul et al. (2003)	a single-center, randomized, parallel-group study	23–45 years old patients with mild persistent asthma	I: 34.3 (5) C: 35.9 (5)	I: 84% C: 80%	I: montelukast 10 mg once daily C: budesonide 400 μg once daily	3 months
Szefler et al. (2007)	a 52-week, open-label, randomized, active-controlled, multicenter study	2–8 years old children with mild asthma or recurrent wheezing	I: 4.7 (1.9) C: 4.6 (2.0)	I: 40.1% C: 38.1%	I: montelukast 4 mg or 5 mg once daily C: budesonide inhalation suspension 0.5 mg once daily	52 weeks
Calhoun et al. (2001)	a multicenter, double-blind, double-dummy, parallel group study	patients aged 15 years and older with asthma	I: 36 (12.75) C: 37 (14)	I: 49% C: 50%	I: montelukast 10 mg once daily (plus placebo fluticasone propionate/salmeterol twice daily) C: fluticasone propionate 100 μg and salmeterol 50 μg twice daily (plus placebo montelukast capsules once daily)	12 weeks
Kanniess et al. (2002)	a placebo-controlled, double-blind, randomised, parallel-group trial	patients with the diagnosis of moderate bronchial asthma	I: 38 (61) C: 43 (54)	I: 50% C: 54.2%	I: montelukast 10 mg once daily C: placebo In the first treatment period, the dose of inhaled corticosteroids (800 μg beclomethasone dipropionate) reduced to 50%, and in the second treatment period, to 25%	two treatment periods of 6 weeks each
Shah et al. (2006)	a double-blind, randomised, controlled trial	asthma patients aged between 18 and 60 years	I: 38.4 (11.2) C: 38.8 (12.0)	I: 20% C: 13.3%	I: montelukast 10 mg once daily in addition to inhaled budesonide 200 μg twice daily C: inhaled budesonide 400 μg twice daily (plus placebo tablets)	8 weeks
Meltzer et al. (2002)	a multicenter, randomized, double-blind, double-dummy, parallel-group study	patients aged 15 years or older with asthma	I: 35.4 (15.5) C: 36.2 (14.5)	I: 49% C: 58%	I: montelukast, 10 mg (plus placebo for fluticasone propionate twice daily) C: fluticasone propionate 88 µg twice daily (plus placebo capsule)	24 weeks
Pearlman et al. (2002)	a 12-week, randomized, double-blind, double-dummy, multicenter study	15 years of age and older patients with persistent asthma	I: 36 (14.75) C: 35 (17)	I: 55% C: 54%	I: montelukast 10 mg once daily (plus placebo fluticasone propionate and salmeterol twice daily) C: fluticasone propionate 100 μg and salmeterol 50 μg twice daily (plus placebo montelukast once daily)	12 weeks
Busse et al. (2001)	a multicenter, randomized, double-blind, double-dummy, parallel-group study	patients aged 15 years or older with a diagnosis of asthma	I: 34.4 (13) C: 35.4 (17)	I: 58% C: 53%	I: montelukast 10 mg (plus placebo twice daily) C: fluticasone propionate 88 µg twice daily (plus placebo capsule)	24 weeks
Noonan et al. (1998)	a double-blind, randomized, three-period, parallel-group study	healthy, nonsmoking chronic asthmatic patients aged 18–65 years	I1: 34.8 (9) I2: 33 (8.7) I3: 31.9 (9.5) C: 36.6 (9.5)	I1: 50% I2: 38.2% I3: 44.4% C: 50.7%	I1: montelukast 2 mg once daily I2: montelukast 10 mg once daily I3: montelukast 50 mg once daily C: placebo	3 weeks
Fish et al. (2001)	two multicenter, randomized, double-blind, parallel-group clinical trials	patients ≥15 years old with persistent asthma	I: 39.5 (12.7) C: 39.9 (12.8)	I: 62% C: 61%	I: montelukast 10 mg once daily C: salmeterol xinafoate powder, 50 μg twice daily	12 weeks

### 3.2 Quality assessment

Risk of bias was prepared for 19 included studies. 3 of studies have high risk of bias, while 16 indicated low risk, as shown on Supplementary Figure S3.

### 3.3 Safety: mental disorders risk

**FIGURE 1:**
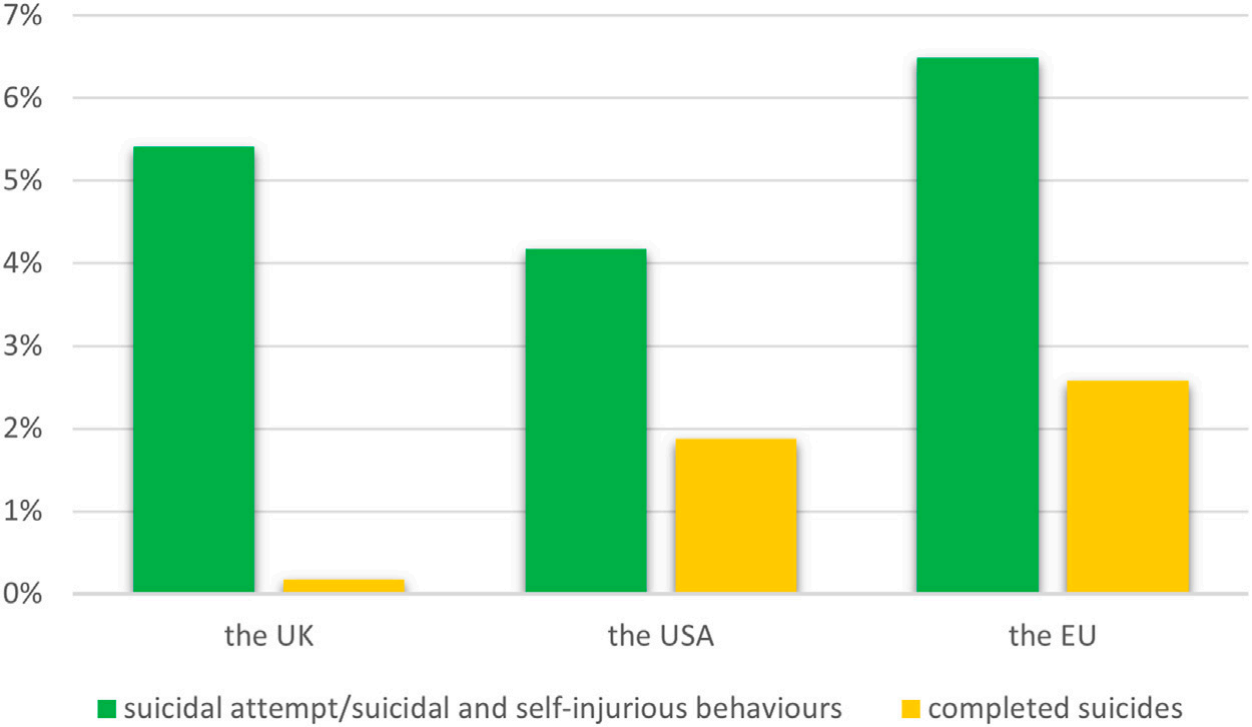
The percentage of suicide-related events among psychiatric disorders after taking of montelukast.

According to data from medical repositories of adverse responses from the United States of America, the European Union (EU) and the United Kingdom, we have shown that among psychiatric disorders after taking montelukast, the % of fatal suicides is 0.17% in the United Kingdom, 1.88% in the United States and 2.57% in the EU (Figure 1).

#### 3.3.1 Anxiety, depression and suicides after montelukast treatment

**FIGURE 2:**
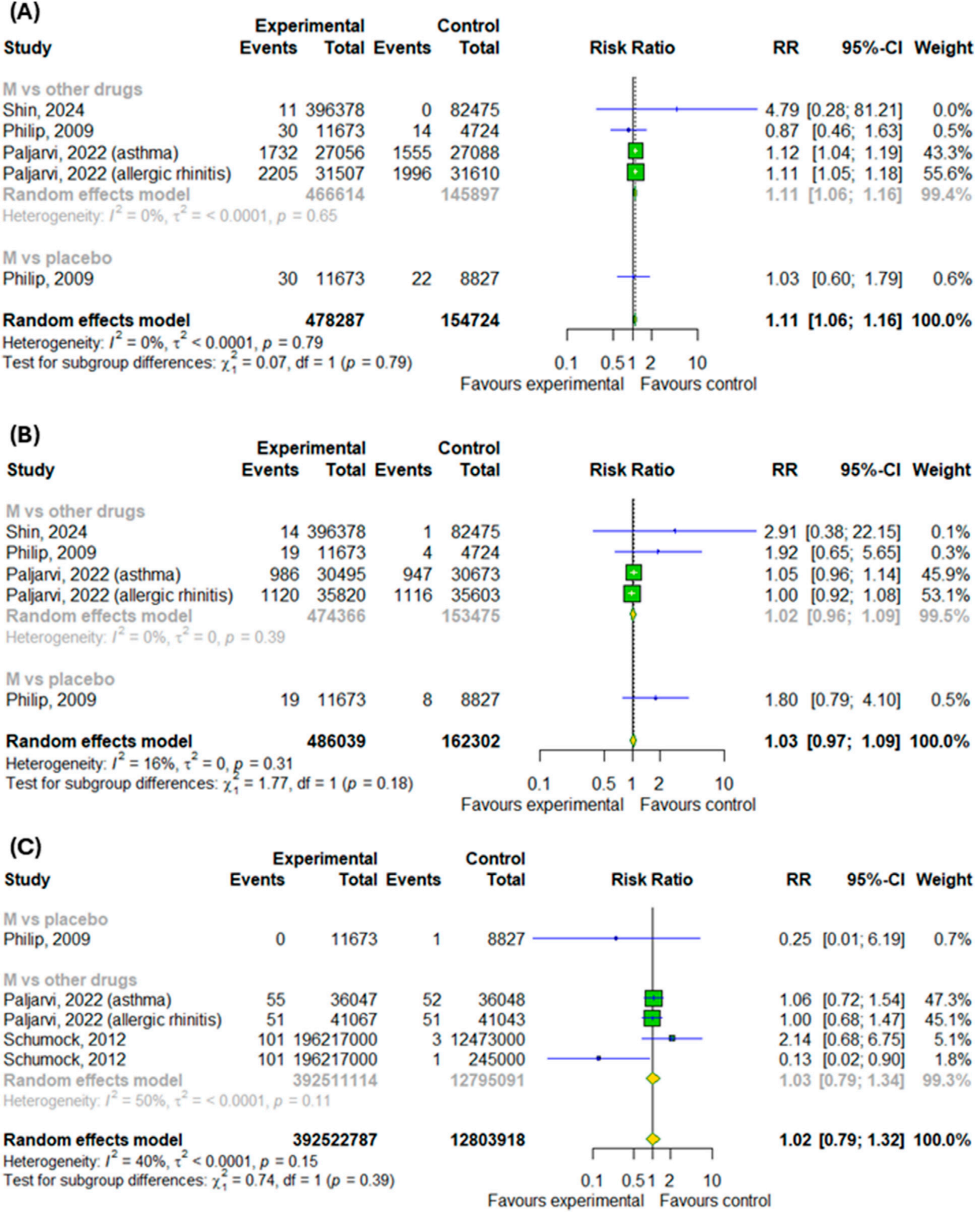
Risk of **(A)** anxiety, **(B)** depression and **(C)** suicidal and self-injurious behaviors, nonfatal self-harm or completed suicides after montelukast treatment compared to control. M–montelukast.

Only 4 studies were included in the subgroup analysis. Overall, montelukast treatment was associated with a higher risk of anxiety by 11% (RR = 1.11; 95% CI [1.06; 1.16]; p < 0.0001, I^2^ = 0%) without differences between subgroups (Figure 2A). However, montelukast treatment didn’t increased the risk of depression as well as suicidal and self-injurious behaviors, nonfatal self-harm or completed suicides (p > 0.05), as showed in Figures 2B,C.

### 3.4 Efficacy

#### 3.4.1 The efficacy of montelukast treatment among patients with asthma and allergic rhinitis

**FIGURE 3:**
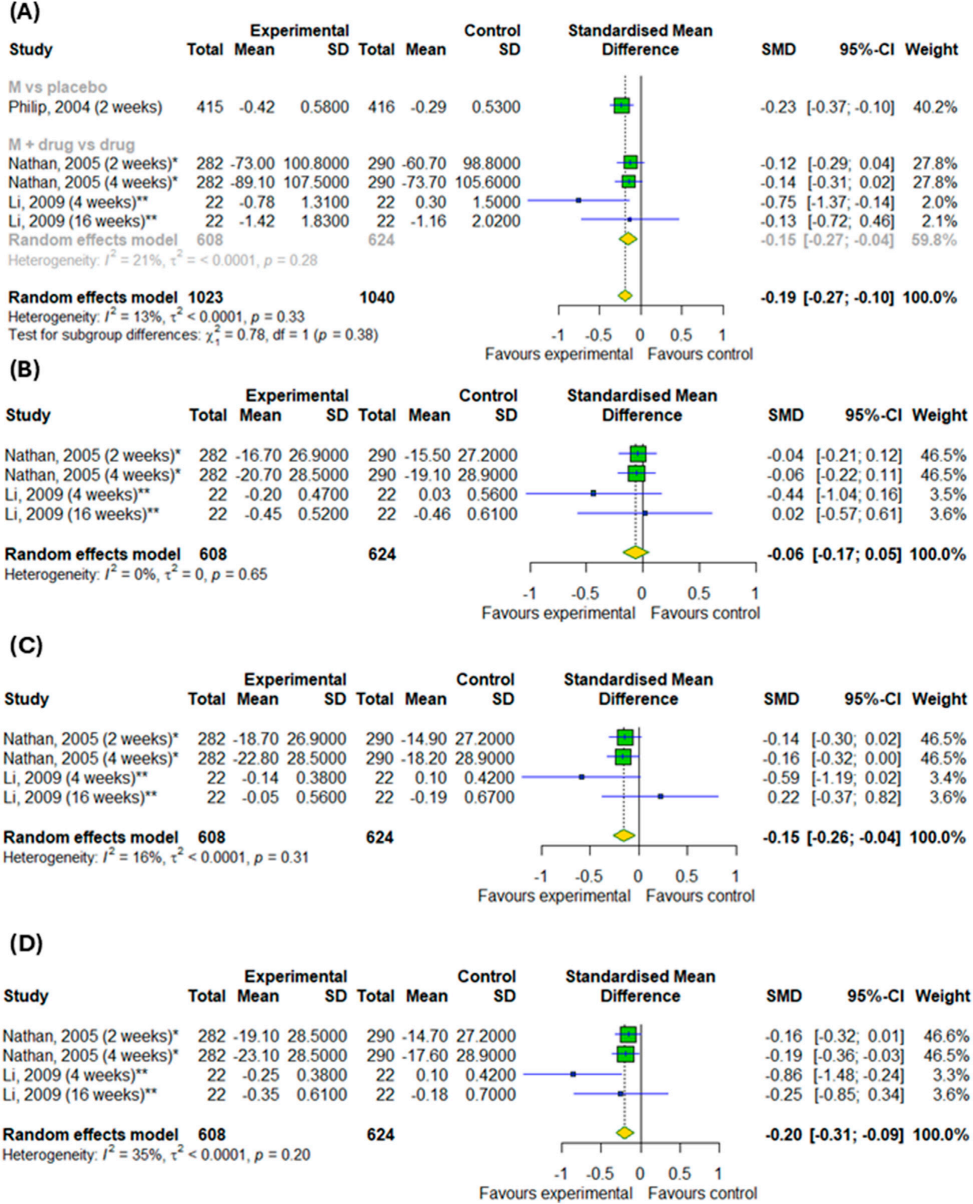
The efficacy of montelukast treatment on the changes in daytime symptoms, such as **(A)** total nasal symptoms or daytime composite symptoms, **(B)** nasal congestion, **(C)** nasal pruritus and **(D)** sneezing in patients with asthma and AR. M–montelukast; *inhaled corticosteroids; **second-generation antihistamines.

**FIGURE 4:**
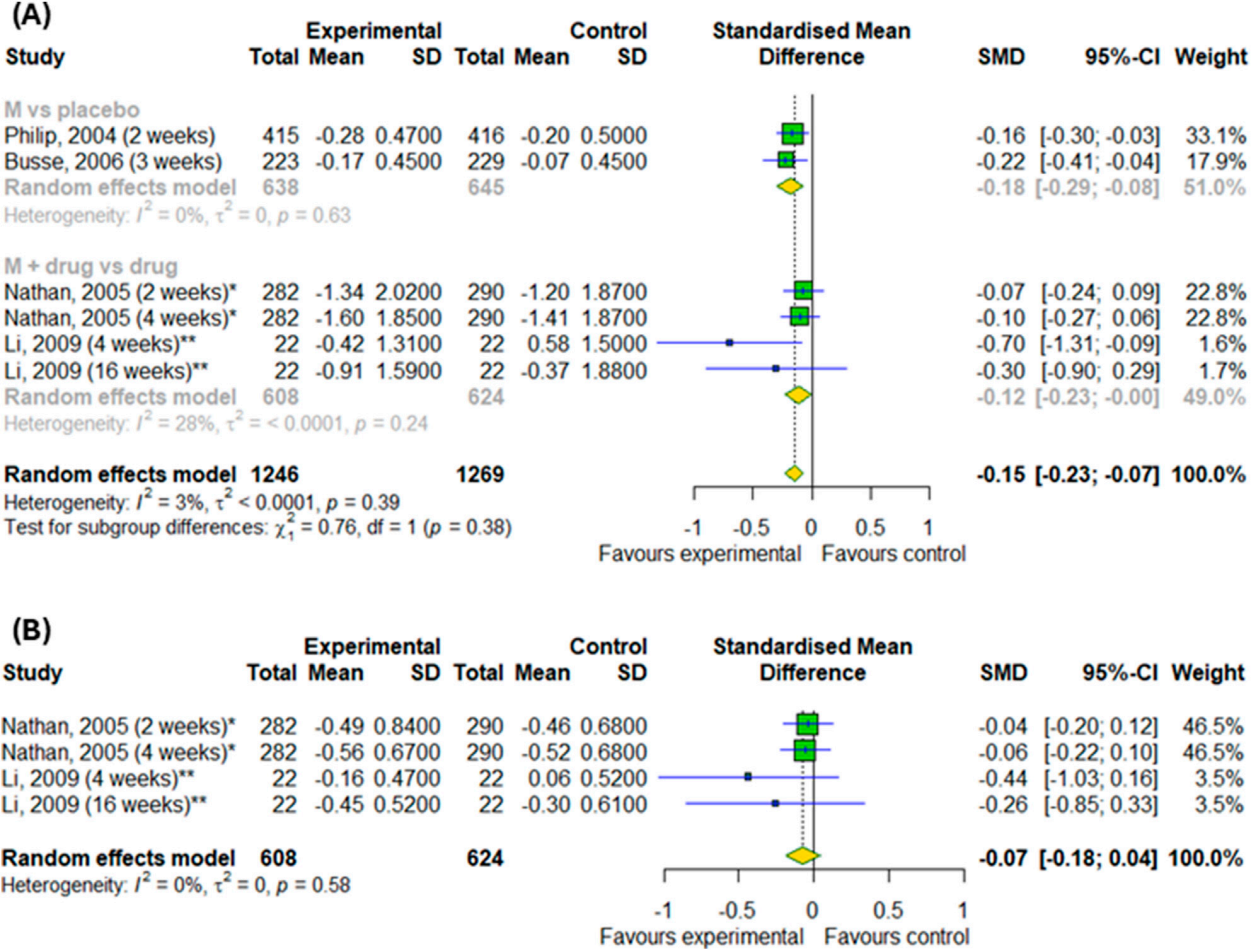
The efficacy of montelukast treatment on the changes in nighttime symptoms, such as **(A)** total nasal symptoms or nighttime composite symptoms and **(B)** nasal congestion in patients with asthma and AR. M–montelukast; *inhaled corticosteroids; **second-generation antihistamines.

We conducted a meta-analysis based on four randomized controlled trials to evaluate the efficacy of montelukast treatment among patients with asthma and allergic rhinitis. Analysis were based on changes in daytime and nighttime symptoms, as shown in Figures 3A–D, as well as Figures 4A,B. The significant differences between the experimental and control groups were observed in daytime symptoms, such as total nasal symptoms or daytime composite symptoms (SMD = −0.19; 95% CI [-0.27; −0.1]); p < 0.0001; I^2^ = 13%) without differences between subgroups, nasal pruritus (SMD = −0.15; 95% CI [-0.26; −0.04]); p = 0.008; I^2^ = 16%) and sneezing (SMD = −0.2; 95% CI [-0.31; −0.09]); p = 0.0005; I^2^ = 35%) and in nighttime symptoms, such as total nasal symptoms or nighttime composite symptoms (SMD = −0.15; 95% CI [-0.23; −0.07]); p = 0.0002; I^2^ = 3%) without differences between subgroups. Although there were no differences in changes in nasal obstruction symptoms during the day or at night (p > 0.05). A noteworthy pattern emerged: montelukast, when used as a standalone medication, proved more effective than a placebo. The efficacy of montelukast was also observed when it was combined with other drugs, as compared to the efficacy of the drugs when used alone.

#### 3.4.2 The efficacy of montelukast treatment among patients with allergic rhinitis

**FIGURE 5:**
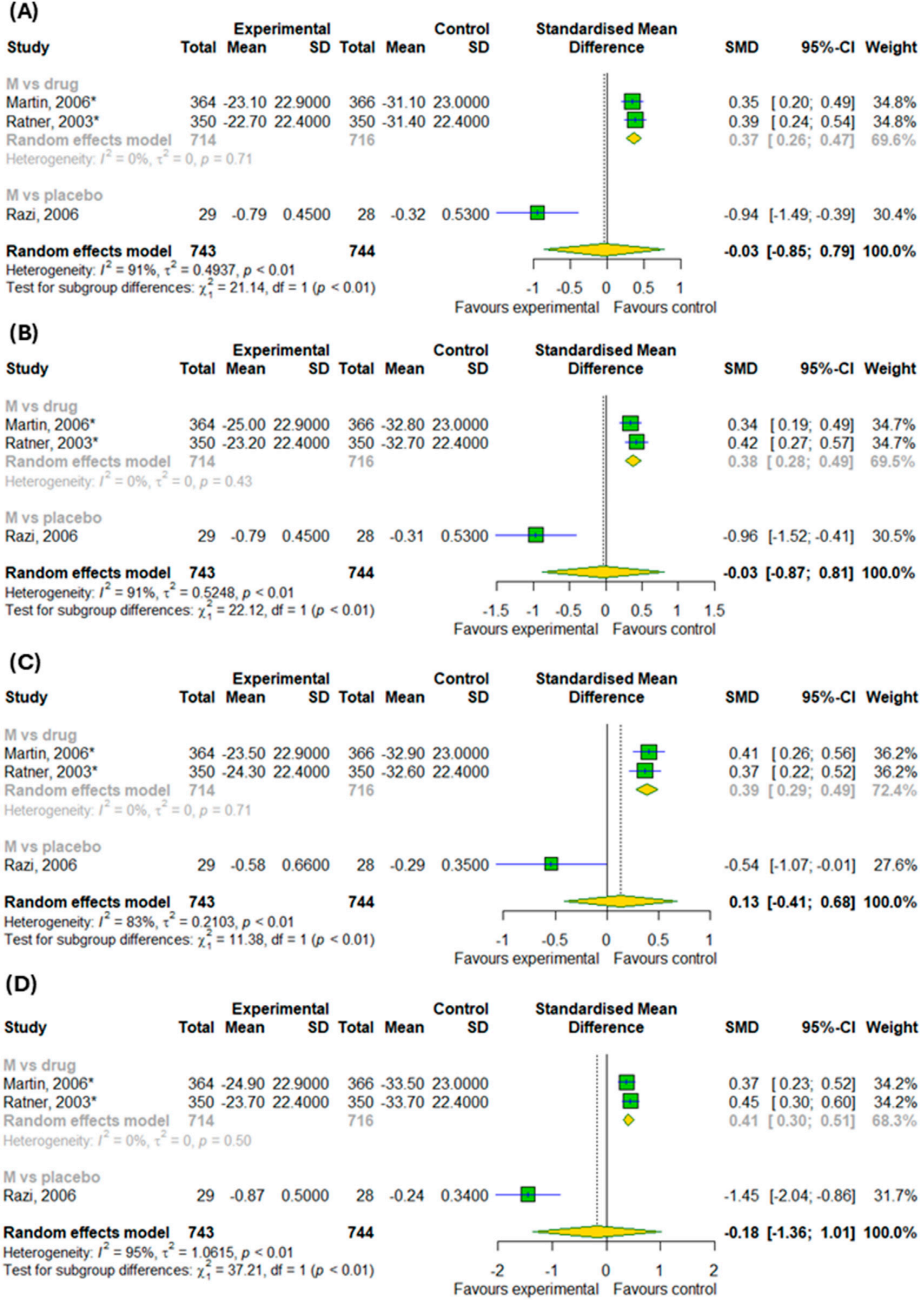
The efficacy of montelukast treatment on the changes in daytime symptoms, such as **(A)** nasal congestion, **(B)** nasal itching **(C)** rhinorrhea and **(D)** sneezing in patients with AR. M–montelukast; *inhaled corticosteroids.

Regarding the efficacy of montelukast treatment in AR patients, the meta-analysis showed no difference in the changes of daytime symptoms in compare to control groups, as illustrated in Figures 5A–D (p > 0.05). However, our subgroup analysis found the differences between subgroups, such as montelukast vs. placebo and montelukast vs. drug, in changes in daytime symptoms, such as nasal congestion (p < 0.0001), nasal itching (p < 0.0001), rhinorrhea (p = 0.0007) and sneezing (p < 0.0001).

**FIGURE 6:**
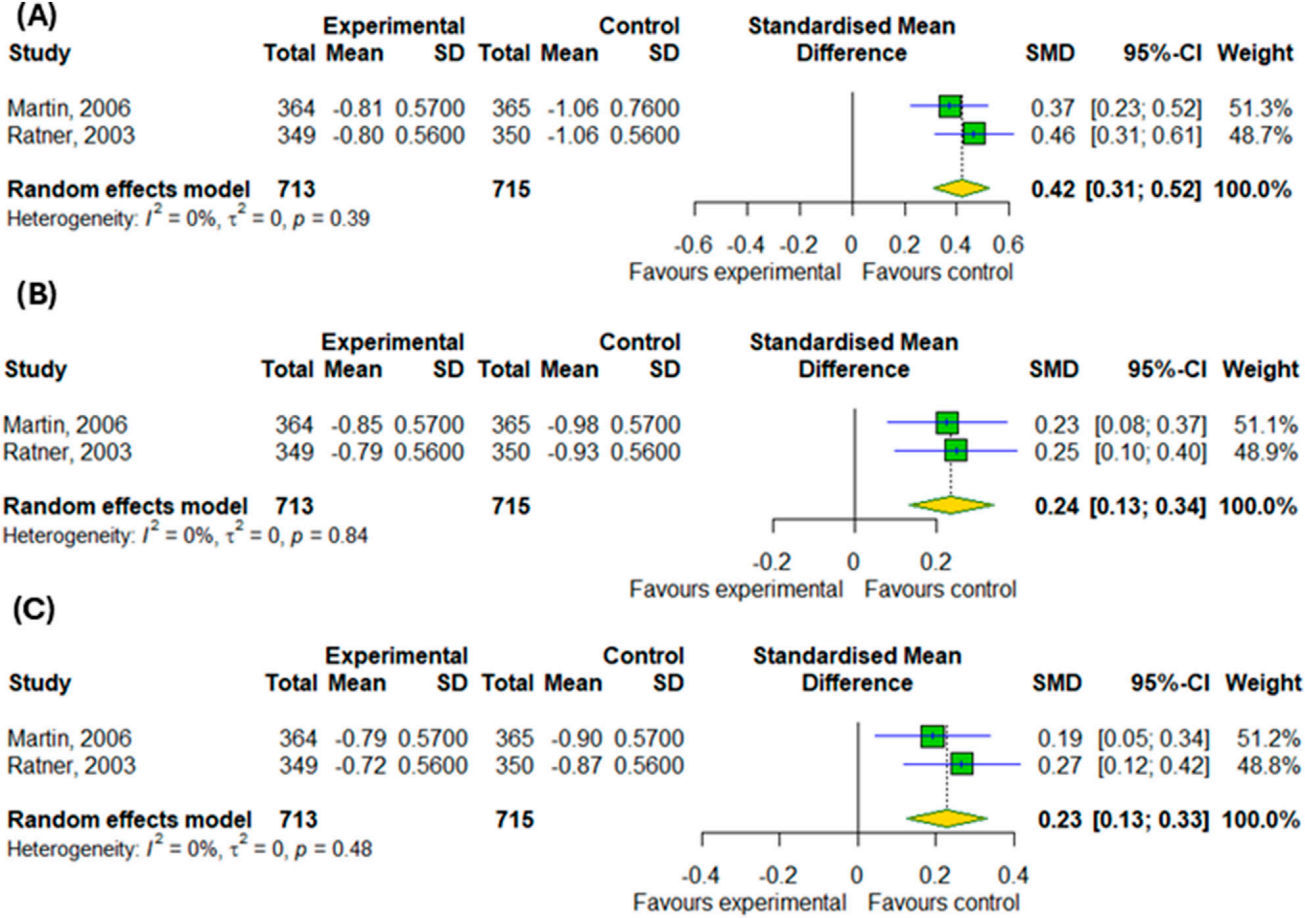
The efficacy of montelukast treatment on the changes in nighttime symptoms, such as **(A)** nasal congestion on awakening, **(B)** difficulty getting to sleep due to nasal symptoms and **(C)** nighttime awakenings due to nasal symptom in patients with AR.

In addition, we also detected differences in the changes in nighttime symptoms, such as nasal congestion on awakening (SMD = 0.42; 95% CI [0.31; 0.52]); p < 0.0001; I^2^ = 0%), difficulty getting to sleep due to nasal symptoms (SMD = 0.24; 95% CI [0.13; 0.34]); p < 0.0001; I^2^ = 0%) and nighttime awakenings due to nasal symptom (SMD = 0.23; 95% CI [0.13; 0.33]); p < 0.0001; I^2^ = 0%) in compare to inhaled corticosteroids, as showed in Figures 6A–C.

**FIGURE 7:**
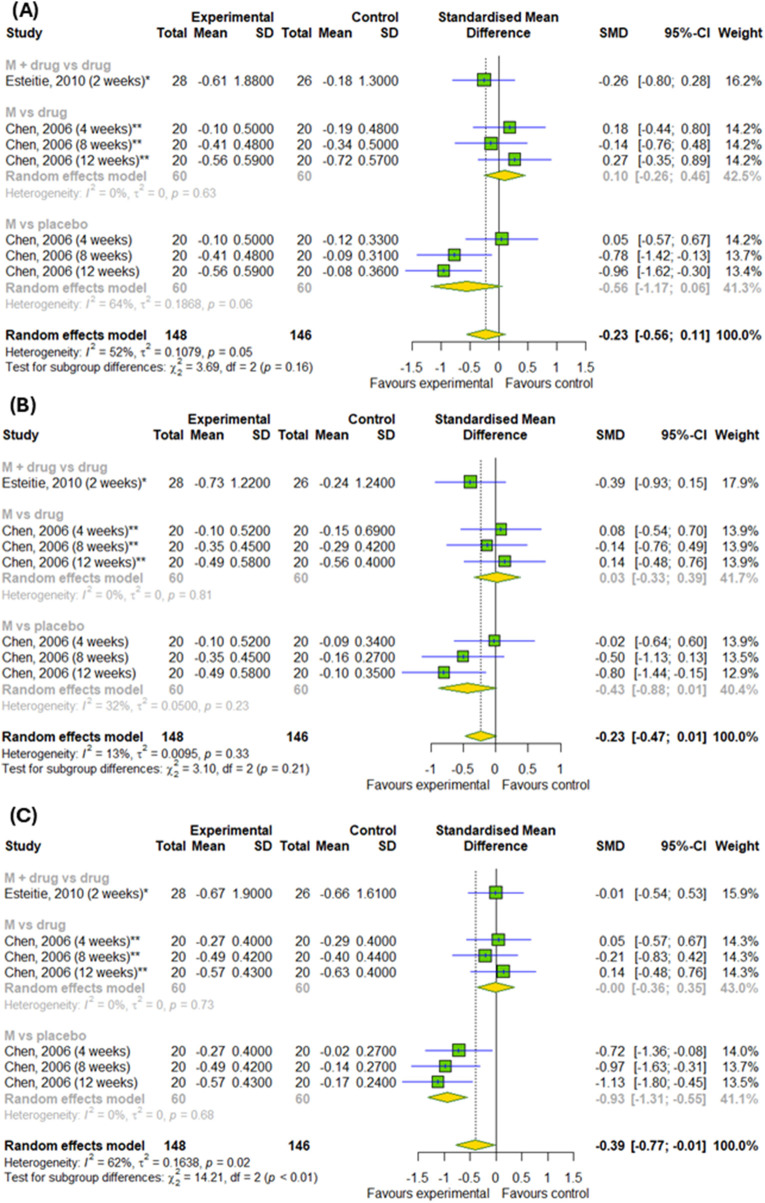
The efficacy of montelukast treatment on the changes in daily symptoms, such as **(A)** sneezing, **(B)** rhinorrhea, **(C)** nasal congestion in patients with AR. M–montelukast; *inhaled corticosteroids; **second-generation antihistamines.

On the last step, we examined the efficacy of montelukast treatment for daily symptoms. There were no differences in rhinorrhea and sneezing symptom changes compared to control groups and there were no differences between subgroups (p > 0.05) (Figures 7A,B). However, we detected differences in the changes in nasal congestion (SMD = −0.39; 95% CI [-0.77; −0.01]); p = 0.046; I^2^ = 62%) between experimental and control groups with differences between subgroups, such as montelukast vs. placebo, montelukast + drug vs. drugs and montelukast vs. drug (p = 0.0008) (Figure 7C).

#### 3.4.3 The efficacy of montelukast treatment among patients with asthma

**FIGURE 8:**
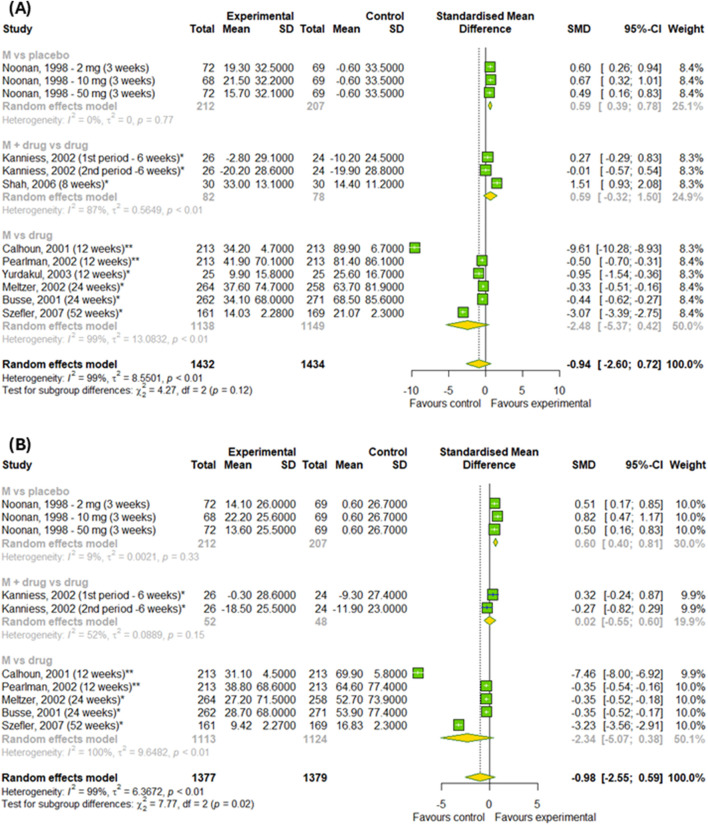
The efficacy of montelukast treatment on the changes in PERF **(A)** in the morning and **(B)** in the evening in patients with asthma. M–montelukast; *inhaled corticosteroids; **second-generation antihistamines.

Figures 8A,B shows the results of meta-analysis conducted on asthma patients showed no difference in changes in PERF in the morning and in the evening results between the experimental and control groups (p > 0.05). However, we detected a difference among subgroups, such as montelukast vs. placebo, montelukast + drug vs. drugs and montelukast vs. drug, in changes in PERF in the evening (p < 0.0001). However, the effect of montelukast was better than placebo (SMD = 0.6; 95% CI [0.4; 0.81]), but worse than active drugs (SMD = −2.34; 95% CI [-5.07; 0.38]).

**FIGURE 9:**
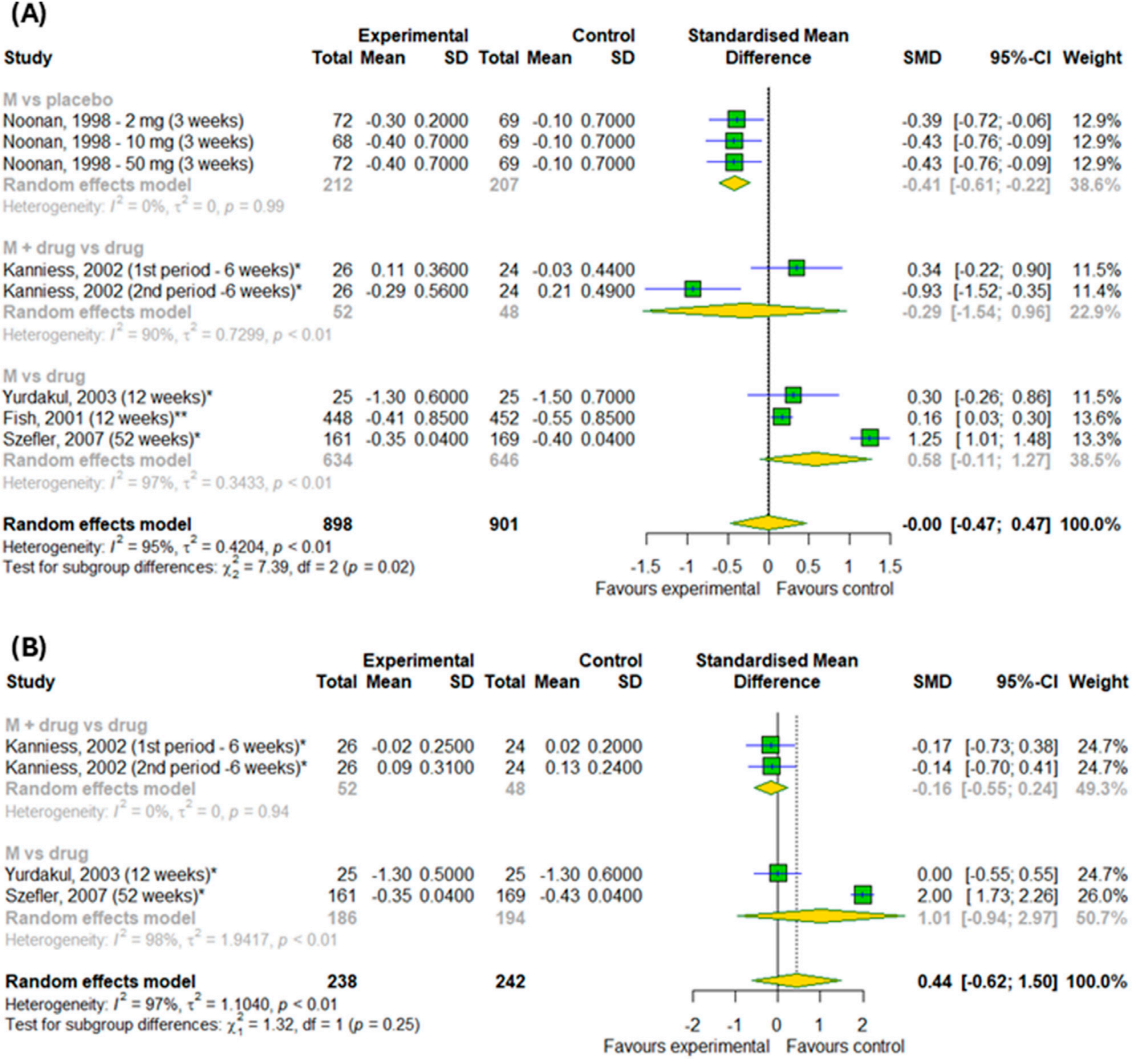
The efficacy of montelukast treatment on changes **(A)** in the daytime and **(B)** nighttime symptoms in patients with asthma. M–montelukast; *inhaled corticosteroids.

In the final stage, we checked the efficacy of montelukast on daytime and nighttime symptoms in patients with asthma (Figures 9A,B). Overall, there was no differences between experimental and control groups (p > 0.05). However, subgroup analysis found the differences between subgroups in changes in daytime symptoms (p = 0.02) – montelukast was better than placebo (SMD = −0.41; 95% CI [-0.61; 0.22]), but worse than active drugs (SMD = 0.58; 95% CI [-0.11; 1.27]).

### 3.5 Publication of bias

We prepared funnel plots (Supplementary Figure S4) and performed Peters’ regression test and Egger’s regression test to calculate publication bias for investigated outcomes. The results showed that there was no evidence of publication bias for the association between montelukast treatment and outcomes, such as changes in PEFR in the morning (p = 0.52) and in the evening (p = 0.4) in patients with asthma; changes in daytime symptoms, such as total nasal symptoms or daytime composite symptoms (p = 0.42), nasal congestion (p = 0.44), nasal pruritus (p = 0.93) and sneezing (p = 0.22) in patients with asthma and AR; nasal congestion (p = 0.07), nasal pruritus (p = 0.12), rhinorrhea (p = 0.056) and sneezing (p = 0.08) in patients with AR, changes in daytime asthma symptoms (p = 0.49) in patients with asthma; changes in nighttime symptoms, such as total nasal symptoms or nighttime composite symptoms (p = 0.11) in patients with asthma and AR, and changes in daily nasal symptoms, such as sneezing (p = 0.56), rhinorrhea (p = 0.98), nasal congestion (p = 0.08) in patients with AR; number of patients with anxiety (p = 0.49), number of patients with depression (p = 0.28) and number of patients with suicidal behaviors (p = 0.64). However, publication bias occur in nighttime symptoms, such as nasal congestion (p = 0.049) in patients with asthma and AR, changes in nighttime asthma symptoms (p = 0.0009) in patients with asthma. Tests for other outcomes could not be calculated because too few studies were included.

## 4 Discussion

In our meta-analysis, we addressed the important topic of whether montelukast treatment is effective and whether it actually affects mental health.

Overall, with regard to neuropsychiatric events in patients with asthma or allergic rhinitis, the meta-analysis of RCTs did not find that montelukast caused an increased risk of their occur (Mou et al., 2023). Although adverse-effect registry data from the EU, United Kingdom and United States show cases of suicide attempts as well as fatalities after montelukast treatment, our further meta-analysis did not show an increased risk of depression or fatal or non-fatal self-harm. In contrast, we have shown that montelukast was associated with a higher risk of anxiety. A large systematic review of 59 studies showed similar results: montelukast is not linked to suicidal or depressive behaviors in patients with asthma. Moreover, neuropsychiatric events, such as anxiety and sleep disorders, are particularly likely to affect older people (Lo et al., 2023). Similarly, a sequence symmetry analysis using data of 11,840 patients from the National Veterans Health Administration found that the initiation of montelukast treatment was not associated with an increased risk of neuropsychiatric events (Fox et al., 2022). In a large study of children and adolescents aged 6–17 years, 26462 of whom used montelukast and 47829 of whom used LABAs, no association was found between montelukast use and an increased risk of neuropsychiatric adverse effects, according to routine clinical practice data (Win et al., 2025). Among children aged between 2 and 5 years with asthma and AR, who started taking combined therapy with montelukast and levocetirizine, 22.1% was developed at least one neuropsychiatric symptom after treatment. Interestingly, improvements in neuropsychiatric outcomes were also observed in this study. 76.5% of patients showed improvement in at least one neuropsychiatric outcome that were present before the treatment (Altaş et al., 2023). On the other hand, a cross-sectional study of 9508 adults (286 of whom were montelukast users) found that montelukast use was associated with an increased risk of depression via multi-faceted mechanisms. Analysis of the Kyoto Encyclopedia of Genes and Genomes (KEGG) enrichment indicates that montelukast primarily acts through multiple pathways, including those involved in endocrine resistance, chemical carcinogenesis, receptor activation, and the estrogen signaling pathway (Yan et al., 2024). Furthermore, a study of montelukast metabolites revealed that montelukast could interfere with the brain’s glutathione detoxification system and disrupt the regulation of various neurotransmitter and neurosteroid pathways. This suggests that montelukast may impact specific processes within the central nervous system (Marques et al., 2022).

There is a correlation between asthma and depression, with specific biological mechanisms and genetic factors playing a key role in their simultaneous occurrence (Tan et al., 2024). Data from observational studies supports the hypothesis that there is a relationship between asthma and suicide-related behaviors, such as suicidal thoughts, attempts, and completion (Iessa et al., 2011). Psychological disorders such as depression are also frequently experienced by patients with AR. Depression has a prevalence rate of between 20% and 40% in AR (Mou et al., 2022). A cross-sectional study showed that patients with AR had higher anxiety and depression scores than those without respiratory symptoms according to Hospital Anxiety and Depression Scale (Rodrigues et al., 2023). Another cross-sectional study based on a population of Korean adolescents also showed a higher chance of despair and suicidal thoughts among AR patients (Cho et al., 2023). A symmetry analysis using three nationwide Danish registries found a weak association between montelukast use and the risk of antidepressant prescription. However, this association was most evident among patients receiving long-term inhaled treatment for chronic asthma. This finding suggests a link between asthma and depression rather than a link between montelukast and depression (Winkel et al., 2018).

Regarding the efficacy of montelukast treatment, we can conclude from our meta-analysis that montelukast is more effective for asthma symptoms and AR than placebo, but it is not more effective than other drugs, such as inhaled corticosteroids or second-generation antihistamines. The overall negative results for some parameters may mean that no advantage of montelukast was found. This means that montelukast has proven clinical efficacy compared to no treatment, but is not the first-line therapy when other drugs with comparable or better efficacy are available. Due to the different treatment regimens in the included studies, we conducted a subgroup analysis. Our subgroup analysis showed no differences in changes in daytime symptoms, such as total nasal symptoms or daytime complex symptoms and nighttime symptoms, such as total nasal symptoms or nighttime complex symptoms compared to the placebo or when added to other drugs for patients with both asthma and AR. However, our subgroup analysis showed differences in changes in daytime symptoms, such as nasal congestion, itching, rhinorrhea and sneezing, for patients with AR. As for daily symptoms in AR patients, differences are shown and overall compared to control groups, as well as differences in subgroups. Moreover, we also detected differences in the changes in nighttime symptoms (nasal congestion on awakening, difficulty getting to sleep due to nasal symptoms and nighttime awakenings due to nasal symptom) in compare to inhaled corticosteroids. Subgroup analysis revealed differences in changes to evening PERF, as well as changes to daytime symptoms among asthmatics. Subgroup analysis showed that the effects of montelukast vary depending on the control group and the symptoms assessed, which should be taken into account when interpreting clinical results.

A meta-analysis evaluating the efficacy of montelukast in treating children with asthma and AR showed similar results. Montelukast was more effective than a placebo at controlling symptoms, but inhaled corticosteroids were more effective (Mayoral et al., 2023). Another meta-analysis produced similar results, showing that inhaled corticosteroids were significantly better than montelukast at preventing severe asthma exacerbations and improving lung function and asthma control in schoolchildren and adolescents with mild to moderate chronic asthma (Castro-Rodriguez and Rodrigo, 2010). Furthermore, the combination of montelukast with levocetirizine is more effective in relieving symptoms of allergic rhinitis than monotherapy among patients with allergic rhinitis with asthma (Shao et al., 2025).

Our study has several limitations. First, all studies included in the mental disorders analysis were retrospective. Such data are subject to underreporting and potential confounding, which limit the ability to infer causality. Therefore, the observed associations should be interpreted with caution, and further prospective studies are needed to confirm these findings. Second, the variables from the studies are presented in different formats and were converted to a single format. Although the conversion methods used are standard in the literature, it cannot be ruled out that they introduced additional variability in the results. Third, the duration of treatment differed among the studies. Shorter observation periods may not reveal subtle therapeutic effects, which limits the possibility of direct comparison of results between studies. Fourth, heterogeneity was high, so we conducted subgroup analyses according to treatment regimens. A possible reason for this may be differences in the baseline characteristics of patients, such as asthma severity or different allergies in people with AR. In addition, different pollen seasons or the duration of treatment and follow-up in individual studies may also contribute to heterogeneity. This suggests that the results should be interpreted with caution, and their generalizability to other populations or clinical settings may be limited. Fifth, our findings are limited in their generalizability to children, as only four studies examined this population. And finally, due to the small number of studies, in some analyses it was not possible to fully assess the risk of publication bias. In summary, although our results provide important information, the above factors should be taken into account when planning further research in this area.

Montelukast is not more effective than other drugs, such as inhaled corticosteroids or second-generation antihistamines, in treatment for asthma and allergic rhinitis. However, it may increase the effectiveness of treatment in combination with other drugs compared to taking drugs alone. This may mean that montelukast should be considered as a complementary or alternative therapy in cases of intolerance or contraindications to standard medications, rather than as a first-line treatment. Furthermore, our study found that montelukast does not increase the risk of depression or suicidal behaviors. These results suggest that the drug can be used safely in most patients in terms of the risk of depressive disorders. At the same time, due to isolated reports of possible adverse effects, it is advisable to monitor patients’ mental health during therapy. Therefore, the use of montelukast should be considered on an individual basis, taking into account the safety profile, alternative therapies and patient preference.

## Data Availability

The original contributions presented in the study are included in the article/Supplementary Material, further inquiries can be directed to the corresponding author.
