# Supplementary material for: Montelukast: risk of mental disorders vs. efficacy–a meta-analysis
Source: Front Pharmacol. 2025 Oct 24;16:1659852. doi: 10.3389/fphar.2025.1659852 (PMC12592120; doi:10.3389/fphar.2025.1659852)
Supplement: Supplementary file 1 [file Supplementaryfile1.docx]

Supplementary Material


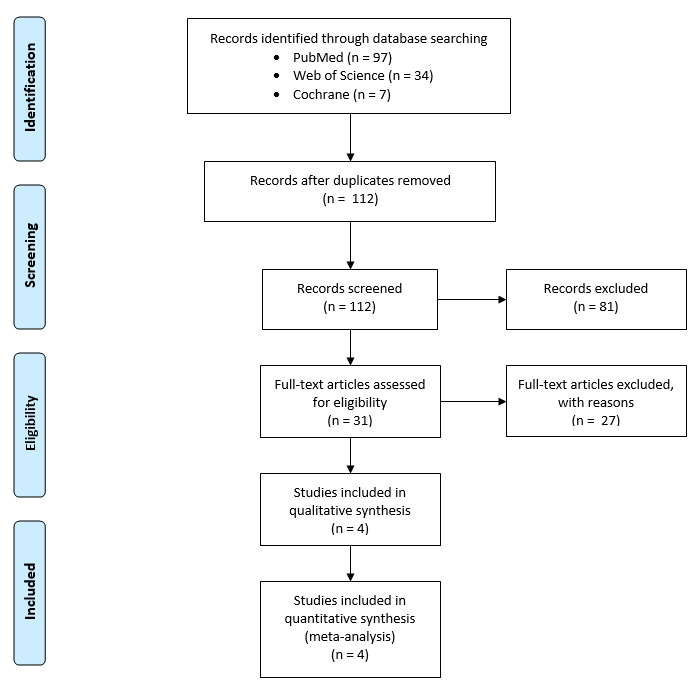


**Supplementary Figure 1. Study selection for mental disorders meta-analysis.**

**
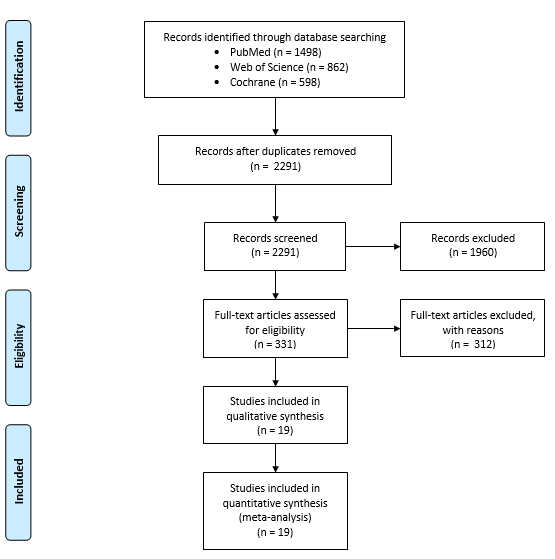
**

**Supplementary Figure 2. Study selection for efficacy meta-analysis.**

**
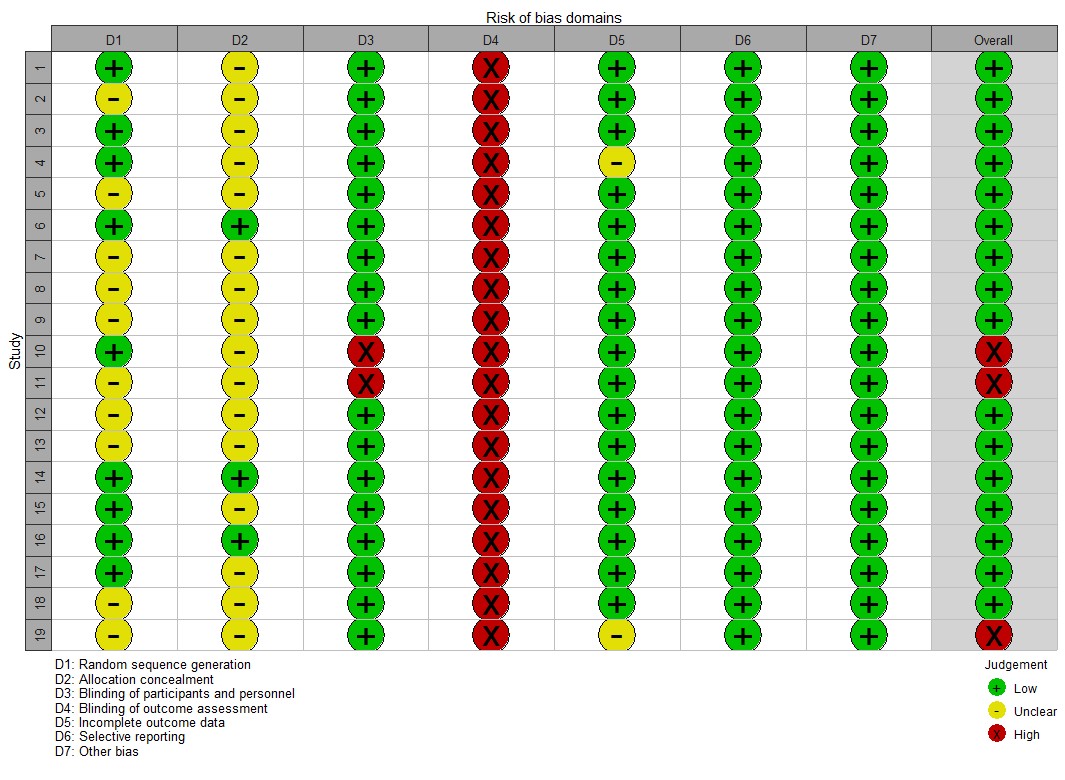
**

**Supplementary Figure 3. Risk of bias of included studies.**

(1) Busse et al., 2006; (2) Nathan et al., 2005; (3) Li et al., 2009; (4) Philip et al., 2004; (5) Martin et al., 2006; (6) Razi et al., 2006; (7) Esteitie et al., 2010; (8) Ratner et al., 2003; (9) Chen et al., 2006; (10) Yurdakul et al., 2003; (11) Szefler et al., 2007; (12) Calhoun et al., 2001; (13) Kanniess et al., 2002; (14) Shah et al., 2006; (15) Meltzer et al., 2002; (16) Pearlman et al., 2002; (17) Busse et al., 2001; (18) Noonan et al., 1998; (19) Fish et al., 2001.


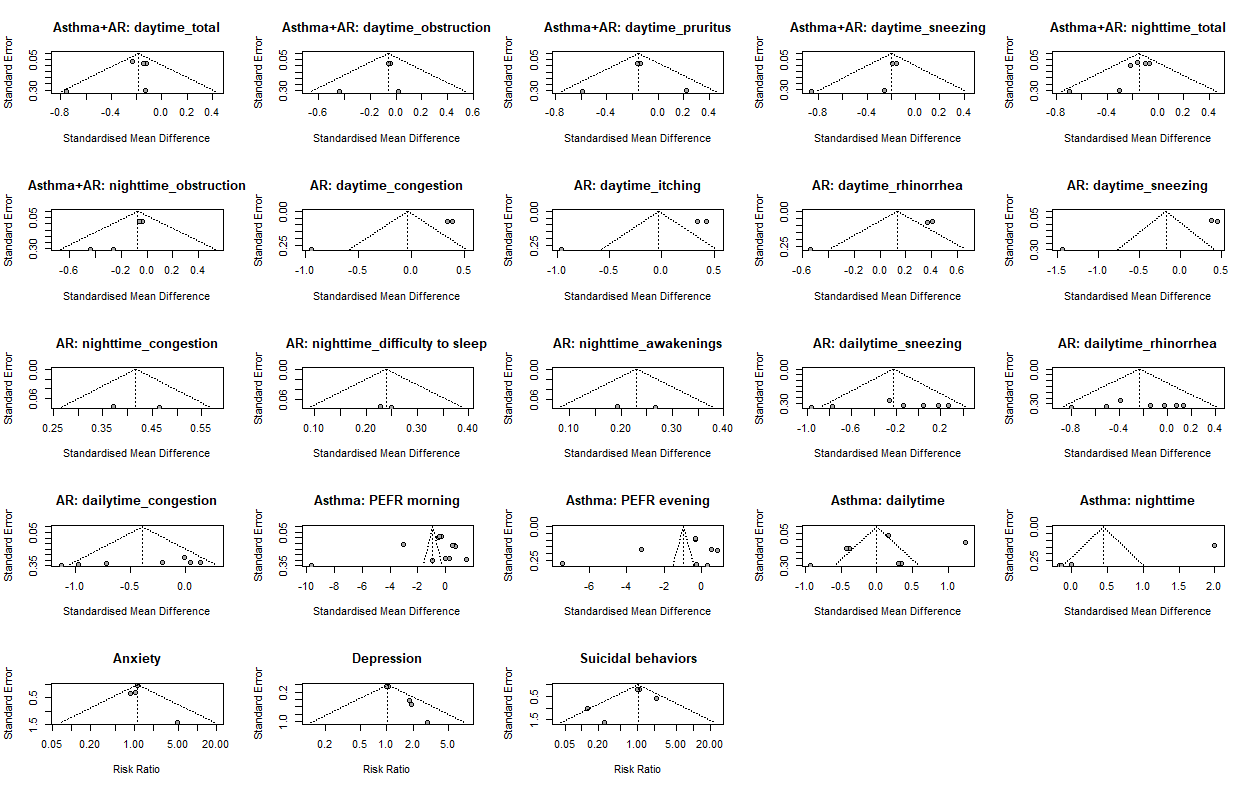


**Supplementary Figure 4. Funnel plots for the association between montelukast treatment and outcomes.**
